# The Impact of Edition Differences in the International Retinoblastoma Classification (IIRC vs. ICRB) on Staging and Globe Salvage Prognosis: Analysis of 642 Eyes

**DOI:** 10.3390/cancers18060895

**Published:** 2026-03-10

**Authors:** Rima Torosyan, Mona Mohammad, Hadeel Halalsheh, Ayat Taqash, Mustafa Mehyar, Ibrahim Al-Nawaiseh, Yacoub A. Yousef

**Affiliations:** 1 Departments of Surgery (Ophthalmology), King Hussein Cancer Centre, Amman 11941, Jordan; torosyanrima@gmail.com (R.T.);; 2 Department of Ophthalmology, Yerevan State Medical University After Mkhitar Heratsi, Yerevan 0025, Armenia; 3 Department of Pediatric Oncology, King Hussein Cancer Centre, Amman 11941, Jordan; 4 Office of Scientific Affairs and Research, King Hussein Cancer Centre, Amman 11941, Jordan

**Keywords:** retinoblastoma, globe salvage, classification system, prognosis

## Abstract

**Purpose:** The objectives of this study were to compare the prognostic performance, staging concordance, and eye salvage prediction of the International Intraocular Retinoblastoma Classification (IIRC) and the International Classification of Retinoblastoma (ICRB), and to assess the clinical impact of definitional discrepancies between the two systems. **Design:** This study used retrospective, observational case series. **Participants:** A total of 642 eyes with intraocular retinoblastoma (Rb) that received conservative management with intent for globe salvage (2003–2024) were assessed. **Methods:** Clinical records and imaging were reviewed for tumor characteristics. Each eye was staged according to both IIRC and ICRB criteria. Prognostic performance for eye salvage was analyzed using odds ratios (OR), the concordance index (C-index), and likelihood ratio χ^2^ tests. **Main Outcome Measures:** The eye salvage rate and concordance between IIRC and ICRB staging were assessed. **Results:** The overall eye salvage rate was 73% (471/642), decreasing significantly with larger tumor size, subretinal fluid (SRF) extent, and more seeding (*p* < 0.0001). The globe salvage was 24% for tumors occupying >50% of the globe compared to 77% for smaller tumors, and 47% for tumors with SRF > 1 quadrant compared to 62% for tumors with SRF ≤ 1 quadrant. Under the IIRC, the salvage rates by group were 98%, 94%, 92%, 51%, and 14% (A–E); under the ICRB, 98%, 94%, 94%, 60%, and 24%. Failure odds (ORs) increased across stages from 2.8 in Group B to 276 in Group E (IIRC) and 145.2 (ICRB). Eighty-seven (14%) eyes were upstaged under the ICRB. Of these, 72 (11%) with tumors > 50% of globe volume shifted from IIRC Group D to ICRB Group E (25% salvage), and 15 (2.3%) with SRF > 3 mm shifted from IIRC Groups B/C to ICRB Group D. The ICRB showed superior discrimination (C-index = 0.824; 95% CI, 0.80250–0.84937) compared with IIRC (C-index = 0.790; 95% CI, 0.768–0.814). **Conclusions:** Both systems accurately predict eye salvage, but definitional variations significantly affect staging and outcome interpretation. The ICRB improves discrimination for advanced tumors by including large tumors in Group E, while the IIRC provides finer stratification in intermediate disease through SRF extent. Integrating these criteria could yield a unified, more prognostically precise classification for Rb.

## Simple Summary

Retinoblastoma is the most common eye cancer in children, and early diagnosis greatly improves the chance of saving the affected eye. Ophthalmologists and ocular oncology specialists use classification systems to stage the disease and estimate the likelihood of eye preservation, but two widely used systems, the International Intraocular Retinoblastoma Classification (IIRC) and the International Classification of Retinoblastoma (ICRB), define advanced disease differently. As a result, the same tumor may be assigned to different stages, which can influence treatment decisions and reported success rates. In this large single-center study of 642 eyes, we compared the IIRC and ICRB to determine how often they assign different stages and how well they predict eye salvage. We found that differences in the definitions of tumor size and subretinal fluid can lead to stage migration and variations in reported outcomes. These findings highlight the need for greater standardization to improve consistency in patient care, research reporting, and international collaboration.

## 1. Introduction

Retinoblastoma (Rb), with an incidence of approximately 1 in 15–18,000 live births, represents the most common primary intraocular malignancy of childhood. In Jordan, Rb is the most common intraocular malignancy in all age groups, with an incidence of 8.2 cases per million children per year among those aged five years or younger (equivalent to approximately one case per 15,620 live births annually) [1,2,3,4].

Survival outcomes are highly favorable when RB is diagnosed at an intraocular stage [5]. However, there is a marked disparity in survival outcomes between low- and middle-income countries and upper-middle- or high-income countries. Children born in low-income settings have a sixteen-fold higher risk of mortality from RB compared with those born in high-income settings [6].

Historically, the first widely used system for intraocular retinoblastoma was the Reese–Ellsworth classification (RE), developed in the 1960s [7] to predict the likelihood of eye salvage following external beam radiotherapy. Although valuable at the time, the RE classification was largely based on treatment outcomes with radiotherapy and became less applicable with the advent of modern chemotherapy and focal therapies in the mid-1990s [7,8].

Among the most widely adopted systems are the International Intraocular Retinoblastoma Classification (IIRC), proposed in 2005 by Linn Murphree [9], and the International Retinoblastoma Classification (IRBC), introduced in 2006 by Shields et al. [10]. Both systems aim to categorize intraocular RB based on tumor size, location, and extent of subretinal fluid, vitreous or subretinal seeding, thereby predicting the likelihood of eye salvage following systemic chemotherapy and focal consolidation therapies.

Despite their shared purpose, notable differences exist between the two systems in terms of classification criteria, prognostic value, and clinical applicability. A precise understanding of these differences is crucial for both clinicians and researchers and can provide insights into their relative strengths and limitations, as well as their impact on clinical decision-making and the expected treatment outcomes.

The probability of ocular salvage is influenced by several tumor characteristics, including tumor size, the presence of subretinal fluid, vitreous seeding, and subretinal seeding [9,10]. The reported salvage rates range from 70% to 100% in eyes with early-stage disease (groups A–C) but decline to approximately 23–50% in advanced stages (groups D and E) [11,12,13]. Furthermore, treatment complexity and burden increase with tumor size and severity. Whereas focal modalities such as transpupillary thermotherapy (TTT) or cryotherapy are generally sufficient for group A tumors, more extensive disease often necessitates combination of advanced interventions, including systemic chemotherapy, intra-arterial chemotherapy (IAC), intravitreal chemotherapy (IVC), and focal consolidation therapy like TTT, cryotherapy and radioactive plaque brachytherapy [14,15,16,17].

The American Joint Committee on Cancer (AJCC) tumor–node–metastasis (TNM) staging system is a standardized framework for all cancers, first incorporating both intraocular and extraocular RB in its 7th edition (2009) [18]. In the 8th edition (2017) [19], the system was revised to predict ocular salvage, metastatic risk, and patient survival more accurately [20].

The purpose of this study is to evaluate and compare the IIRC and ICRB, focusing on their similarities and differences, the impact of using different classification systems on the distribution of tumors across groups, and their predictive value for eye salvage in each group. Highlighting these differences will underscore how classification choice can influence clinical decision-making, research interpretation, treatment planning, and eye salvage prognosis while emphasizing the need for clinicians worldwide to ‘speak the same language’ when describing disease grade and prognosis.

## 2. Methods

This is a retrospective case series approved by the Institutional Review Board at King Hussein Cancer Center (25KHCC261). It involves 642 eyes diagnosed with RB between January 2003 and December 2024 at the King Hussein Cancer Center (KHCC). Data was acquired from patients’ medical charts, Ret Cam photos, pathology, and radiology reports. Diagnosis was based on characteristic clinical features supported by multimodal imaging, including ultrasonography, Ret Cam photography, and orbit and brain MRI. Outcome measures included demographics, laterality, staging at diagnosis, tumor features, management, management outcome, and eye salvage. All tumors were restaged according to IIRC [9] and ICRB [10]. Tumors were assessed according to size, site, presence and extent of subretinal fluid (SRF), presence and severity of subretinal and vitreous seeding, and the presence of Group E features based on each classification. Treatment outcomes were categorized as globe salvage or treatment failure, with failure defined as eyes requiring secondary enucleation or external beam radiation therapy (EBRT).

### 2.1. Inclusion and Exclusion Criteria

Inclusion criteria: This study included patients diagnosed with intraocular Rb who had initial staging with a detailed clinical description and Ret Cam images at diagnosis and who were classified according to both the IIRC and the ICRB and were treated at KHCC. Patients were included if sufficient clinical and imaging data was available to determine the diagnosis and classification status under both the IIRC and ICRB systems. Only eyes that received conservative therapy as primary treatment with the aim of eye salvage were included.

**Exclusion criteria:** Patients who had received any form of treatment before classification at our institute, as such interventions could alter tumor classification and preclude accurate assessment of globe salvage. Additionally, those who underwent primary enucleation or primary EBRT, patients with extraocular RB, and those with insufficient follow-up data (less than one year) were excluded from this study.

### 2.2. Tumor Features, Definitions, IIRC and ICRB Classification Systems, and Treatment Modalities

We reviewed RetCam images and clinical drawings for all eyes at the time of diagnosis and documented each tumor feature. We then classified the tumors according to IIRC [9] and ICRB [10] criteria for Rb. Restaging according to both IIRC and ICRB criteria was independently performed by two experienced ocular oncologists. In cases of discrepancy, consensus was reached through joint review.

Tumor size was classified into 3 categories: ≤3 mm, >3 mm but involving ≤half of the globe, and >half of the globe, as determined clinically, by B-scan ultrasonography, and/or magnetic resonance imaging. According to the IIRC [9], tumor size per se is not explicitly incorporated into the classification scheme except for Group A, in which tumors must be ≤3 mm in the greatest dimension; however, in the ICRB [10] system, a tumor occupying more than half of the globe is considered Group E disease.

Subretinal fluid was classified into 3 categories based on its extent: involving ≤3 mm, >3 mm but ≤one quadrant, and >one quadrant.

In the IIRC, SRF extending no more than 5 mm from the tumor base is considered Group B, SRF involving ≤one quadrant corresponds to Group C, whereas fluid SRF > one quadrant of the retina is categorized as Group D, but according to the ICRB, SRF > 3 mm from the tumor base is considered a Group D feature.

Tumor seeding was classified according to location as subretinal or vitreous, and its appearance: seedings were considered focal when either the fine local aggregates of tumor cells were present without a diffuse pattern (IIRC) and/or they were ≤3 mm from RB (ICRB), and massive when diffuse aggregates were observed (IIRC) and/or they were >3 mm from RB (ICRB).

According to the IIRC, eyes are classified as Group E when they are anatomically or functionally destroyed by the tumor and therefore are unsalvageable. This includes the presence of one or more Group E features such as irreversible neovascular glaucoma, massive intraocular hemorrhage, aseptic orbital cellulitis, a tumor located anterior to the anterior vitreous face, a tumor in direct contact with the lens, diffuse infiltrating retinoblastoma, or advanced ocular atrophy such as phthisis or pre-phthisis. The presence of any of these features (IIRC-E features) was defined as a potentially unsalvageable eye in this study. In contrast, the ICRB defines Group E disease as the presence of an extensive RB occupying more than 50% of the globe or the occurrence of one or more severe clinical findings (neovascular glaucoma, opaque ocular media secondary to hemorrhage in the anterior chamber, vitreous, or subretinal space, as well as evidence of invasion into critical structures such as the post-laminar optic nerve, choroid exceeding 2 mm in thickness, sclera, orbit, or anterior chamber). For this analysis, eyes classified as Group E according to the ICRB were further subdivided into two subgroups: E1 and E2. This provisional subdivision was based on tumor size. Tumors occupying more than half of the globe were categorized as E1, while eyes meeting other ICRB Group E criteria were categorized as E2. Although this subclassification is not part of the original ICRB, it was applied in the current study to evaluate the impact of extensive intraocular tumor on the prognostic performance of the staging system.

For most patients with intraocular RB, we used a combination chemotherapy regimen of CVE (carboplatin, vincristine, and etoposide). Each CVE cycle was repeated every 4 weeks for a total of 6–8 cycles according to the patient’s condition and tumor status. Ocular oncology follow-up was provided with examination under anesthesia before every cycle of chemotherapy and every 4 weeks thereafter. Fundus images were obtained using a RetCam system (RetCam II, Clarity Medical Systems, and RetCam, Natus Medical Incorporated; Pleasanton, CA, USA). Combination focal therapy was applied as needed, as TTT and/or triple freeze–thaw cryotherapy (MIRA CR 4000). IAC, IViC, subtenon chemotherapy, and I-125 radioactive plaque brachytherapy were used as second-line treatment options for tumor recurrence or for residual tumor activity. For this study, we defined treatment failure as the need for EBRT or enucleation.

### 2.3. Statistical Methods

The primary endpoint was globe salvage. Multivariate Logistic Regression models were used to estimate odds ratios (ORs) and 95% confidence intervals (CIs) for the prognostic power of IIRC and ICRB groups. The discriminatory ability of each staging system was assessed using the concordance index (C-index) [21] and prognostic homogeneity was evaluated with likelihood ratio χ^2^ tests; higher values for each indicated better performance. Odds ratios (ORs) for treatment failure across groups were calculated using logistic regression, with Group A as the reference. *p* values were determined by the Chi-squared test, with significance set at *p* ≤ 0.05. All analyses were conducted using SAS software (v9.4, SAS Institute Inc., Cary, NC, USA).

## 3. Results

A total of 642 eyes for 578 patients with intraocular Rb were included in this analysis. Of these, 125 (19%) eyes were for patients with unilateral disease and 517 (81%) eyes for patients with bilateral Rb. Eye globe salvage was higher in bilateral cases, with 415 of 517 eyes (80%) retained, compared with 56 of 125 eyes (45%) among unilateral cases (*p* < 0.0001).

### 3.1. Tumor Features

Based on the anatomical location, 169 (26%) eyes were categorized as intraretinal tumors (Groups A and B), 394 (61%) eyes were categorized as extraretinal tumors (Groups C and D), SRF was seen in 156 (24%) eyes, subretinal seeds in 302 (47%) eyes, vitreous seeds in 255 (40%) eyes, 72 (11%) eyes had large tumors involving more than half of the globe, and 7 (1%) eyes had advanced unsalvageable tumors (Table 1).

### 3.2. Association Between Tumor Features and Eye Salvage

Eyes without SRF achieved the highest salvage rate of 81%, which fell to 47% in eyes with SRF involving more than one quadrant. Eyes with no subretinal seeds had 86%, while eyes with massive subretinal seeding had a 50% salvage rate. Eyes with no vitreous seeds had an 85% salvage rate, while eyes with massive vitreous seeds had a 38% salvage rate (Table 1). Small tumors smaller than 3 mm had a 97% salvage rate, while tumors filling more than half of the eye globe had a 24% salvage rate. The presence of features of unsalvageable eye globe (IIRC-E features) had the worst eye salvage rate (14%), as shown in Table 1.

### 3.3. Eye Salvage Based on IIRC and ICRB Grouping

**Figure 1:**
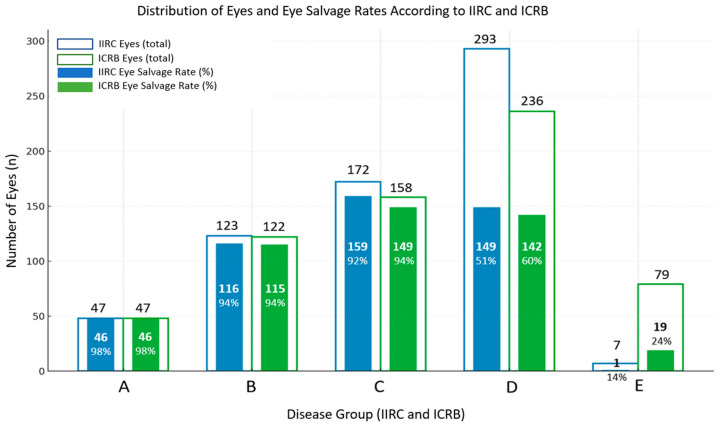
The curve illustrates the distribution of eyes across the different groups according to the International Intraocular Retinoblastoma Classification (IIRC) and the International Classification for Retinoblastoma (ICRB). It also presents the corresponding eye salvage rates for each group within both staging systems. Group A includes small tumors (<3 mm) confined to the retina away from critical structures; Group B includes larger tumors or tumors closer to the macula or optic disc without seeding; Group C includes tumors with localized vitreous or subretinal seeding; Group D includes tumors with diffuse vitreous or subretinal seeding with extensive intraocular disease; Group E includes very advanced disease with features indicating minimal chance of globe salvage, and Group E1 represents the subset of Group E eyes characterized by a large intraocular tumor occupying more than half of the globe.

Eye salvage outcomes according to each staging system showed differences in certain groups (Table 2). Under the IIRC, the salvage rates were 98% for Group A (46 of 47), 94% for Group B (116 of 123), and 92.4% for Group C (159 of 172). Salvage dropped markedly in Group D to 51% (149 of 293), and in Group E to 14% (1 of 7). The ICRB system produced similar high salvage rates in early stages: 98% in Group A, 94% in Groups B and C, but differed in advanced groups. Of 236 eyes classified as Group D, 142 were salvaged (60%), representing a higher salvage proportion than IIRC Group D, while the 79 eyes classified as Group E had a salvage rate of 24% (19), higher than the IIRC Group E but with a much larger case load (Figure 1, Table 2). According to both the IIRC and the ICRB, Groups C and D had significantly higher odds of treatment failure compared with Groups A and B, and Group E had the highest treatment failure risk. The overall effect of the IIRC grouping was highly significant (*p* < 0.0001) (Table 3).

Logistic regression confirmed the prognostic strength of both classification systems using Group A as the reference. The IIRC demonstrated the following odds ratios (OR) for failure: 2.8 for Group B, 3.7 for Group C, 44.5 for Group D, and 276 for Group E. The overall model effect was highly significant (likelihood ratio Chi-squared = 176.79; *p* < 0.0001), and the model demonstrated good discriminative ability (C-index = 0.790; Bootstrap 95% CI, 0.768–0.814). Similarly, the ICRB system revealed strong predictive value across all groups: ORs of 2.8 for Group B, 2.8 for Group C, 30.4 for Group D, 145.2 for Group E, 138 for Group E1 (large tumors > 50% of the eye), and 276 for E2 tumors. Subclassification of Group E into E1 and E2 was exploratory; due to the extremely small number of E2 cases (*n* = 1), no meaningful statistical inference could be drawn, and this subgroup is presented descriptively. This exploratory subdivision was introduced to evaluate whether tumor bulk alone accounts for the prognostic distinction attributed to ICRB Group E. Although not part of the original classification, this analysis was designed to inform future refinement of advanced intraocular staging criteria. The overall model effect was highly significant (likelihood ratio Chi-squared = 207.37; *p* < 0.0001), with strong discriminative ability (C-index = 0.824; 95% Bootstrap CI, 0.802–0.850) (Table 3).

### 3.4. Distribution of Eyes Based on IIRC and ICRB and Stage Migration

Cross-tabulation of the IIRC and the ICRB systems revealed notable discrepancies between the two different staging systems. Among 642 eyes, 47 were classified as Group A, and 122 were consistently categorized as Group B in both systems. Only one eye (0.2%) was classified as Group B by IIRC but upgraded to Group D according to the ICRB criteria. While 172 eyes were assigned to Group C under the IIRC, 14 (2.2%) of these were reclassified as Group D by the ICRB due to differences in the assessment of subretinal fluid (under the IIRC, tumors with SRF ≤ 5 mm were categorized as Group B, whereas the ICRB classified such cases as Group D). SRF > 3 mm frequently triggered an upgrade to Group D in the ICRB, whereas the IIRC would place these cases in Group B (if ≤5 mm without seeding) or Group C (if >5 mm but involving <1 quadrant of retina) (Table 4).

In advanced disease stages, the IIRC classified 293 (46%) eyes as Group D and 7 (1%) as Group E, whereas because the ICRB defines large tumors (>50% of globe volume) as Group E, it reclassified 72 (11%) of the IIRC Group D eyes into Group E, resulting in 236 (37%) eyes in Group D and 79 (12%) in Group E (Table 4).

Overall, 555 eyes (86%) retained the same grade across both systems, including all Group A eyes, most Group B eyes, and a proportion of Groups D and E. Grade migration occurred in 87 eyes (14%), all of which were upgraded under the ICRB system (i.e., the ICRB grade was higher than that in the IIRC). The most frequent reclassification involved 72 eyes (11.2%) migrating from IIRC Group D to ICRB Group E. This reallocation had prognostic implications: eyes reclassified into Group E1 exhibited a globe salvage rate of 25%, notably lower than that of IIRC Group D (51%) yet higher than the poor outcomes seen in IIRC Group E (14%).

## 4. Discussion

Both editions of the international classification for retinoblastoma (IIRC and ICRB) demonstrated strong ability to predict globe salvage following chemoreduction and focal consolidation therapy. However, clinically meaningful stage migration was observed in 14% of eyes, all of which were upgraded under the ICRB system. Most discrepancies (83%) occurred between IIRC Group D and ICRB Group E and were primarily attributable to the inclusion of large tumor size (>50% of globe volume) as a defining criterion for Group E in the ICRB. Such definitional differences complicate cross-study comparisons and undermine efforts toward a unified staging “language” in the literature. Consistent with prior reports [11,12,13,22], smaller tumors, absence of subretinal or vitreous seeds, and minimal subretinal fluid (SRF) were strongly associated with higher salvage rates in our cohort, with globe salvage exceeding 80–90% in Groups A–C and declining to 20–50% in Groups D–E. Although both systems were designed to stratify prognosis in the era of systemic chemotherapy and focal therapies, differences in key definitions—particularly tumor size and SRF thresholds—directly influenced group allocation and reported outcomes.

The IIRC, introduced by Murphree [9], emphasizes descriptive features such as focal versus massive seeding and defines Group E based on irreversible anatomic or functional destruction (e.g., neovascular glaucoma, phthisis, anterior segment invasion), without incorporating tumor bulk alone. In contrast, the ICRB, proposed by Shields et al. [10], applies stricter quantitative criteria for seeding and classifies tumors occupying more than half of the globe as Group E, reflecting their poor salvage potential.

In our cohort, 11% of eyes had tumors involving >50% of the globe. These eyes were classified as Group D by IIRC but as Group E by ICRB. Importantly, this subgroup demonstrated a 25% salvage rate—lower than IIRC Group D overall (51%) but higher than IIRC Group E (14%). Consequently, reclassification under ICRB increased reported salvage for Group D (60% vs. 51%) while also increasing salvage for Group E (24% vs. 14%) because the ICRB Group E population included large tumors with intermediate salvage potential. Thus, differences in Group D and E outcomes between systems largely reflect redistribution of large tumors rather than intrinsic prognostic superiority. Studies employing ICRB may therefore report more favorable Group D outcomes and comparatively higher Group E salvage than studies using IIRC. A similar definitional shift was observed for SRF. Eyes with SRF ≤ 1 quadrant but >3 mm were classified as ICRB Group D and IIRC Group C. This subgroup demonstrated a 74% salvage rate—intermediate between IIRC Group C (92%) and ICRB Group D (60%)—again illustrating how threshold definitions affect stage assignment and reported outcomes. Only one eye migrated from IIRC Group B to ICRB Group D, precluding meaningful interpretation.

Although the ICRB demonstrated a statistically higher C-index than the IIRC (0.824 vs. 0.790), the magnitude of difference reflects incremental improvement in model discrimination rather than a transformative change in bedside management. The primary advantage lies in refined research stratification and comparability rather than immediate therapeutic decision shifts.

Our findings expand upon prior reports. Novetsky et al. [23,24] observed group discrepancies in 5.2% of eyes, whereas in our larger cohort of 642 eyes, discrepancies affected 14%, predominantly between IIRC Group D and ICRB Group E. Shields et al. [12] demonstrated long-term globe salvage rates using ICRB of 96% (A), 91% (B), 91% (C), 71% (D), and 32% (E), comparable to our results (98%, 94%, 94%, 60%, and 24%, respectively). Similarly, CHLA studies using IIRC reported 94% salvage for Group B [25] and 68% 60-month globe survival for Group D [24], compared with 94% and 51% in our cohort. Collectively, this data confirms that both systems provide robust prognostic stratification, though through different structural frameworks: IIRC offers descriptive clinical clarity, whereas ICRB emphasizes quantitative precision.

The controversy surrounding large tumor size remains unresolved. Singh et al. [26] reported higher primary enucleation rates among eyes upstaged from Group D to Group E under ICRB (30% vs. 11%) and proposed further subdivision of Group E. Conversely, Kim et al. [13] argued that tumor bulk alone does not justify Group E designation, noting clearer distinction between Groups D and E using IIRC and similar high-risk pathology rates among reclassified eyes. Importantly, AJCC-OOTF registry data [27,28,29] demonstrated that increasing tumor size significantly correlates with metastatic mortality and high-risk histopathologic features. Five-year survival declined from 99% in small tumors to 83% in the largest category (*p* < 0.001), and tumors occupying >2/3 of the globe showed significantly increased the odds of high-risk pathology (OR 3.3–4.1) [29]. This data supports tumor bulk as a biologically meaningful prognostic factor, although it is not explicitly incorporated into the AJCC 8th edition staging [19,29].

The strengths of this study include its large sample size (642 eyes) and consistent management at a single tertiary center. Limitations include the retrospective restaging of eyes and the exclusion of patients with insufficient follow-up or primary enucleation, which may underestimate advanced disease. The exclusion of eyes undergoing primary enucleation may lead to overestimation of salvage rates in advanced groups and limits generalizability of absolute salvage outcomes. However, because both staging systems were applied to the same selected cohort, the comparative analysis between IIRC and ICRB remains internally valid.

Future prospective multicenter studies should evaluate both globe salvage and survival outcomes while aiming to integrate the descriptive clarity of IIRC with the quantitative precision of ICRB into a unified, biologically coherent classification system.

## 5. Conclusions

In conclusion, both the IIRC and ICRB provide valuable frameworks for stratifying intraocular RB; however, important differences exist that can impact clinical decision-making and reported outcomes. Definitional discrepancies, particularly regarding tumor size and SRF, can lead to divergent group assignments and variable reported eye salvage rates. These inconsistencies reinforce the need for a unified classification system, which would optimize treatment planning and strengthen international collaboration by ensuring that clinicians “speak the same language” when reporting outcomes. Based on our findings, a unified classification system should integrate the quantitative precision of the ICRB with the practical clinical clarity of the IIRC. Specifically, we propose that: (1) Large tumors occupying >50% of the globe should be formally recognized as an advanced category given their substantially reduced salvage rate (~25%) and established association with high-risk pathology and metastatic mortality. (2) However, such cases should be distinguished from anatomically destroyed eyes with irreversible complications (e.g., neovascular glaucoma, phthisis), preserving a separate ‘unsalvageable’ category. (3) SRF thresholds should adopt a consistent quantitative cutoff (e.g., >3 mm) while preserving descriptive clarity regarding quadrant involvement. (4) Seeding definitions should combine quantitative distance criteria with descriptive pattern characterization. Such a structured hybrid approach would improve prognostic homogeneity while maintaining clinical usability.

## Figures and Tables

**Table 1:** Distribution of demographics, clinical features, and corresponding eye salvage rates in intraocular retinoblastoma.

Category	Subcategory	Number	%	Salvage (*n*)	Salvage (%)	*p* Value
Laterality	Unilateral	125	20	56	45	<0.0001
	Bilateral	517	80	415	80	
Total Eyes	-	642	-	471	73	
Anatomical Location ICRB	Intraretinal tumors (Groups A and B) *	169	26	161	95	<0.0001
	Extraretinal Tumors (Groups C and D) **	394	61	291	74	
	Advanced (large tumors > 50%) ***	72	11	18	25	
	Advanced (unsalvageable with E eye features) ****	7	1	1	14	
Subretinal Fluid	No	486	76	392	81	<0.0001
	<1 quadrant	37	6	23	62	
	>1 quadrant	119	18	56	47	
Subretinal Seeds	No	340	53	291	86	<0.0001
	Focal	173	27	115	66	
	Massive	129	20	65	50	
Vitreous Seeds	No	387	60	330	85	<0.0001
	Focal	138	22	97	70	
	Massive	117	18	44	38	
Tumor Size	<3 mm	96	15	93	97	<0.0001
	3 mm—½ globe	468	73	359	77	
	>½ globe	78	12	19	24	
Features of E Eye	No	635	99	470	74	<0.0001
	Yes	7	1	1	14	

* Well-defined tumors confined to the retina; ** tumors with focal extension into subretinal or vitreous spaces; *** tumors occupying more than 50% of the globe clinically or by imaging; **** eyes with profound structural damage due to tumor growth or secondary complications (IIRC-Group E).

**Table 2:** Distribution of 642 eyes with intraocular retinoblastoma and eye salvage rates according to IIRC and ICRB classifications.

Classification	Group	Number	Percentage (%)	Eye Salvage	Salvage (%)
IIRC	A	47	7	46	98
	B	123	19	116	94
	C	172	27	159	92
	D	293	45	149	51
	E	7	1	1	14
ICRB	A	47	7	46	98
	B	122	19	115	94
	C	158	24	149	94
	D	236	37	142	60
	E	79	12	19	24

IIRC: International Intraocular Retinoblastoma Classification [9]. ICRB: International Classification for Retinoblastoma [10].

**Table 3:** Prognostic performance of IIRC, and the ICRB staging systems for eye salvage.

	Concordance Indices			Likelihood Ratio χ^2^		
	C-Index		Bootstrap 95% CI			
IIRC	0.790		0.76841–0.81413	(176.788) < 0.0001		
ICRB	0.824		0.80250–0.84937	(207.3722) < 0.0001		
			**Odds Ratio Estimates**			
	Eye Salvage %	Effect	Odds Ratio (OR)	95% Wald Confidence Limits		*p* value
**IIRC System**						
A	46 (98%)	-	-	-	-	-
B	116 (94%)	B vs. A	2.800	0.335	23.392	0.3419
C	159 (92%)	C vs. A	3.737	0.476	29.324	0.2098
D	149 (51%)	D vs. A	44.451	6.051	326.561	0.0002
E	1 (14%)	E vs. A	275.966	15.194	>999.999	0.0001
	(C and D) vs. (A and B)		10.222	4.900	21.327	<0.0001
	(E) vs. (A and B)		120.714	12.943	>999.999	<0.0001
**ICRB System**						
A	46(98%)	-	-	-	-	-
B	115 (94%)	B vs. A	2.800	0.335	23.392	0.3419
C	149 (94%)	C vs. A	2.778	0.343	22.510	0.3384
D	142 (60%)	D vs. A	30.447	4.128	224.548	0.0008
E	19 (24%)	E vs. A	145.245	18.752	>999.999	<0.0001
E1 *	18 (25%)	E1 vs. A	137.983	17.735	>999.999	<0.0001
	(C and D) vs. (A and B)		7.121	3.382	14.994	<0.0001
	(E) vs. (A and B)		63.534	26.417	152.800	<0.0001

Abbreviations: IIRC, International Intraocular Retinoblastoma Classification, ICRB, International Classification of Retinoblastoma. * This classification of Group E eyes into E1 was not mentioned in the ICRB; however, it is mentioned here to show the impact of making large tumors filling more than half of the globe on the prognostic performance of the staging system and the reported eye salvage rate.

**Table 4:** Comparative distribution and stage migration of intraocular retinoblastoma cases classified by IIRC and ICRB (*n* = 642 eyes) and eye salvage (*n*, %).

	**ICRB**						
		A	B	C	D	E	Total
**IIRC**	A	**47** (46, 98%)	0	0	0	0	47 (46, 98%)
	B	0	**122** (116, 95%)	0	1 (1, 100%)	0	123 (116, 94%)
	C	0	0	**158** (149, 94%)	**14** (10, 71%)	0	172 (159, 92%)
	D	0	0	0	**221** (131, 59%)	**72** (18, 25%)	293 (149, 51%)
	E	0	0	0	0	**7** (1, 14%)	7 (1, 14%)
	Total	47 (46, 98%)	122 (116, 95%)	158 (149, 94%)	236 (142, 60%)	79 (19, 24%)	642 (471, 73%)

## Data Availability

The data presented in this study are available on request from the corresponding author.

## Abbreviations

The following abbreviations are used in this manuscript:

RB	Retinoblastoma
KHCC	King Hussein Cancer Centre
RE	Reese–Ellsworth
IIRC	International Intraocular Retinoblastoma Classification
ICRB	International Retinoblastoma Classification
EBRT	External beam radiation therapy
IAC	Intra-arterial chemotherapy
IViC	Intravitreal chemotherapy
SRF	Subretinal fluid
